# Procedure for Determining Dimensional Allowances for PPE Using 3D Scanning Methods

**DOI:** 10.3390/ijerph19042397

**Published:** 2022-02-19

**Authors:** Joanna Szkudlarek, Grzegorz Owczarek, Marcin Jachowicz, Bartłomiej Zagrodny

**Affiliations:** 1 Department of Personal Protective Equipment, Central Institute for Labour Protection-National Research Institute, 90-133 Lodz, Poland; growc@ciop.lodz.pl (G.O.); majac@ciop.lodz.pl (M.J.); 2 Department of Automation, Biomechanics and Mechatronics, Lodz University of Technology, 90-924 Lodz, Poland; bartlomiej.zagrodny@p.lodz.pl

**Keywords:** personal protective equipment, dimensional allowances, ergonomic design, occupational health and safety

## Abstract

The article describes the importance of dimensional allowances, which are a consequence of the use of personal protective equipment (PPE) for work safety. The method of 3D scanning was proposed for determining the dimensional allowances which has been preliminary validated. Two geometric solids (a cylinder and a cuboid) were used to approximate the minimum space around the person using PPE. The solids are a simplified representation of the silhouette of a human subject performing activities in a confined work environment. They also correspond to the typical shapes of access openings and confined spaces, reflecting the real working conditions of welders, firefighters, mine rescuers, and other rescue teams. A detailed analysis of dimensional allowances for a full welding PPE set is provided. Based on the adopted parameters: the dimensions of the body, the base area and the volume, the differences in the dimensions of the body of a person dressed in underwear and in PPE were compared. The results of the presented studies indicate a significant role of dimensional allowances in interactions between persons wearing PPE and the work environment. The results are planned to be implemented in a new anthropometric atlas of human’s measures used for ergonomic design.

## 1. Introduction

The construction features of many technical devices, including personal protective equipment (PPE), are modeled using computer aided design (CAD) systems. PPE, tools, machines, and work environments can be designed without the participation of human subjects in a cost-effective manner with the application of anthropometric databases. Expanding such databases to include information about dimensional allowances for PPE will facilitate the ergonomic design of the aforementioned elements.

Anthropometric measurements constitute an important element of the input data in the production of protective clothing, gloves, and footwear, as well as head, eye, and face protection devices, ensuring their optimum fit both in terms of user comfort and safety. Moreover, as regards the work environment, anthropometric data are used for the design of ergonomic workplaces, machines, and tools, taking into account the use of PPE. Thus, it is necessary to take into consideration the space occupied by the workers wearing PPE.

Many countries around the world have conducted anthropometric research in the area of developing and updating national sizing systems used for the design of casual and occupational clothing, including functional clothing, as well as work environments. Some of the most notable databases are the Anthropometric Survey of US Army Personnel (ANSUR) [1], PEOPLESIZE 2008 [2], the Internationaler Anthropometrischer Datenatlas: Schriftenreihe der Bundesanstalt für Arbeitsschutz Fb 587 [3], Sizegermany [4], the World Engineering Anthropometry Resource (WEAR) [5], the Civilian American and European Surface Anthropometry Resource (CAESAR) [6,7], SizeUK [8], and Sizing Up Australia [9].

In Poland, anthropometric studies have been conducted and published, amongst others, by the Industrial Design Institute [10,11], the Polish Academy of Sciences [12], and the Central Institute for Labor Protection (CIOP) [13]. The Industrial Design Institute research has been focused on anthropometric measurements of different age groups of the Polish population for the needs of industry. The crucial goal of the studies conducted by the Polish Academy of Sciences has been to elucidate the biological conditions of the working-age population, depending on social and environmental factors. The CIOP research has concentrated on the needs of those who design work environments. The aforementioned institutions have engaged in cooperation to develop a comprehensive dataset of information useful in designing workplaces, including anthropometric and biomechanical data, as well as workspace parameters and safety dimensions. These efforts have given rise to the Atlas of Human Measures [13].

Advances in science and technology have now made it possible to conduct anthropometric measurements by analyzing images from contactless 3D scanners, which is more time efficient as compared to manual methods. 3D scanner images are used to generate highly accurate spatial data complete with a 3D view of the body known as an “avatar” for virtual design. For many years now, 3D scanning technology has brought benefits in the area of ergonomic design of protection devices. Contemporary research mostly concentrates on rendering the shape of the human body with the aim of improving the fit of PPE [14,15,16,17,18,19] as researchers determine the so-called internal dimensional allowances to improve the fit of PPE in accordance with the anthropometric traits of individual users.

**Figure 1:**
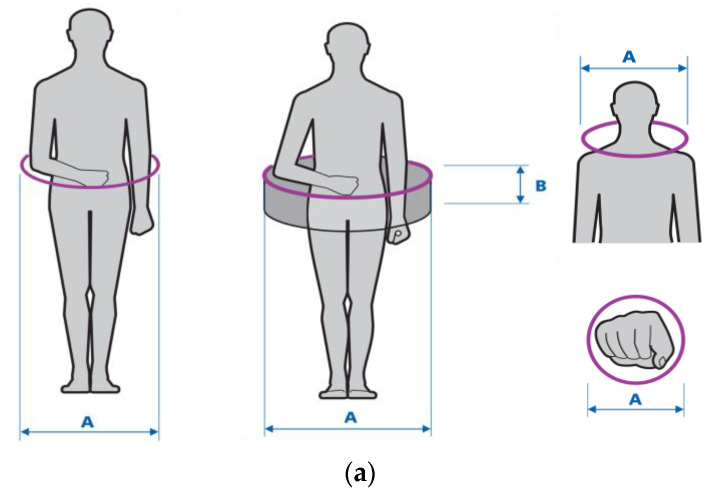

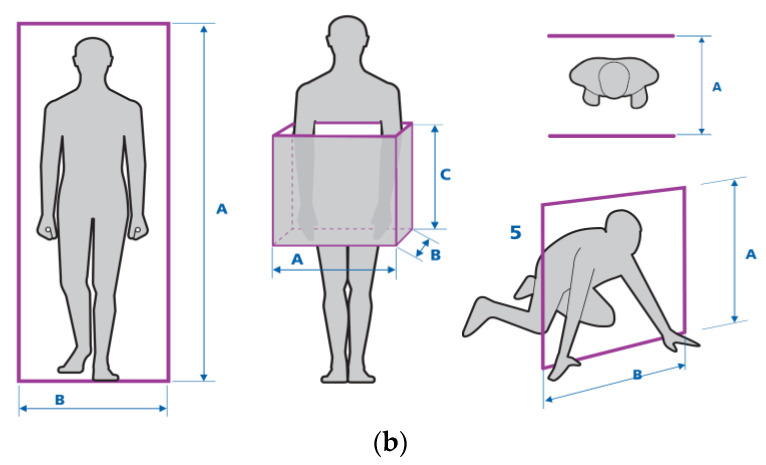
Illustration of geometric figures modeling/approximating the actual shapes of confined spaces: (**a**) circle/cylinder; (**b**) rectangle/cuboid [13].

It should be noted that the dimensional allowances discussed in this publication have a different meaning as they increase the external dimensions of PPE users. In other words, they represent the values that should be added to anthropometric measures to reflect the air spaces between the body and the PPE plus the total dimensions of the PPE itself. These allowances are crucial for the safety of limited accessibility entrances/exits and the safety of work in confined and enclosed spaces. The cross-sections of such spaces are usually circular (e.g., sewer and storm drain manholes) or rectangular (e.g., ventilation ducts). Thus, the geometric solids approximating those spaces are cylinders and cuboids, respectively. The need to classify confined spaces into those two categories was already indicated in the Atlas of Human Measures [13] from 2001. Figure 1 presents geometric figures modeling the actual shapes of confined spaces entered by workers (whether by the entire human body or in part) during occupational activity.

The classification of confined and enclosed workspaces by passage cross-section refers to the actual working conditions of technical and rescue teams. Confined spaces include tunnels, chambers, boilers, pressure containers, sewer and ventilation pipes, and ducts, where work is hindered by restricted movement and limited ability to perform tasks [20,21,22]. Such conditions by themselves may constitute a hazard to workers due to the risk of getting stuck. In such cases, the knowledge of PPE-related dimensional allowances is important in that it can be used to facilitate rescue operations and plan evacuation routes for the injured.

The terms used in the context of occupational safety in the European standards include “height and width allowances” and “minimum gaps” (safety distances). The standards EN 547-1:1996+A1:2008 [23] and EN 547-2:1996+A1:2008 [24] address the need to add height or width allowances (or both) to anthropometric data in determining the dimensions of passages and access openings. These allowances take into account cases such as fast walking, running, wearing heavy footwear and work clothing, as well as the use of PPE.

In turn, the standard EN 349:1993+A1:2008 Safety of Machinery – Minimum gaps to avoid crushing of parts of the human body [25] shows the relationship between dimensional allowances and safety distances protecting against mechanical hazards. 

**Figure 2:**
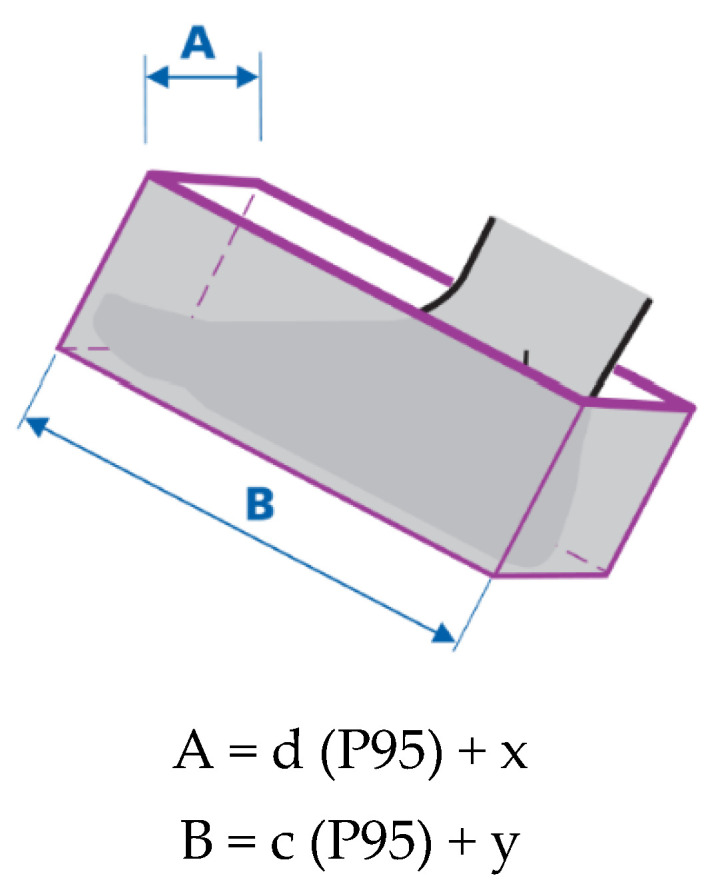
Dimensional allowances for foot width (x) and length (y) used in designing openings for one foot to access to ankle bone according to EN 547-2:1996 +A1:2008. A—Opening width, B—Opening length, d—Foot breadth, c—Foot length, P95—95th percentile [13,24].

In the standard EN 547-2:1996+A1:2008 [24], a cuboid is used to describe the dimensions of the human body, e.g., a foot within an access opening (the minimum space required for a foot to enter an opening up to the ankle bone, taking into consideration width and length allowances). An illustration showing directions for the design of access openings for one foot is given in Figure 2.

Width and length allowances are calculated from the formula given in Figure 2. The formula uses information about the human foot’s width and length for the 95th percentile of the population. In the case of access opening for forefoot-operated control actuators, width and length allowances should be added to the anthropometric measures specified in EN 547-3:1996+A1:2008 [26], if need be. 

In addition to presenting a method for determining dimensional allowances using geometric solids, the aim of this work is to systematize knowledge about dimensional allowances, distinguishing between those that are required for PPE comfort and fit and those that define the space occupied by workers wearing PPE, and promoting their recognition as an important element in designing PPE, tools, and work environments according to ergonomic principles.

The terminology concerning dimensional allowances for PPE, which was introduced in the standards EN 349 and EN 547, was first used in Poland in the Atlas of Human Measures by A. Gedliczka [13]. The Atlas contained the results of research conducted by the CIOP-PIB, where it was suggested that allowances should be added to anthropometric measurements to improve work ergonomics. The Atlas of Human Measures also noted that workspace and safety dimensions are associated with the external dimensions of the human body. According to the authors of the 2001 Atlas, data on increments in the dimensions of users wearing PPE are important for work in hazardous conditions.

Welding is often performed in conditions of low accessibility and hindered movement, that is, in confined spaces (such as ducts, containers, the double hulls and compartments of ships, the interiors of technical structures), where workers may be exposed to multiple hazards at the same time. Given the above, the improvement of the occupational safety and comfort of welders is of interest to OSH specialists.

The hazardous and harmful factors in welders’ work environments include those of a mechanical, physical, and chemical nature, and in particular electrocution, extremely high temperatures and fire, molten metal splashes, hot workpieces (potentially causing limb, face, and neck burns), metal particles, sparks, and drops (risk of eye and face injury). Other hazards include the risk of mechanical injury to the arms, legs, and the whole body (due to falling structural elements, slips, etc.), harmful radiation, and work under poor lighting conditions, dust, poisonous and noxious chemical compounds, and explosion risks [27,28].

Dimensional allowances for PPE are defined as the distance between any point located on the external surface of PPE and the point on the user’s body located under it in the normal direction. Thus, a dimensional allowance for PPE is the difference between an anthropometric measurement of the subject wearing PPE (whether a single item or a kit) and a measurement of that subject without PPE [29].

The objective of the study was to develop a method for automatic determination of the maximum external dimensions of the silhouette of workers wearing the PPE required for the occupational tasks being performed by them. The scope of the study encompasses:

- identification of the main anthropometric landmarks needed for the determination of the external dimensions of the human body;
- development of an optimum method for automated sizing, involving geometric solids for the description of the human silhouette, i.e., solids with the lowest volume that can circumscribe the human body wearing underwear and PPE, in order to establish the difference in their dimensions, referred to as a dimensional allowance;
- the systematization of terminology and making a distinction between dimensional allowances that affect PPE fit and those that affect human-workspace interactions.

## 2. Materials and Methods

**Figure 3:**
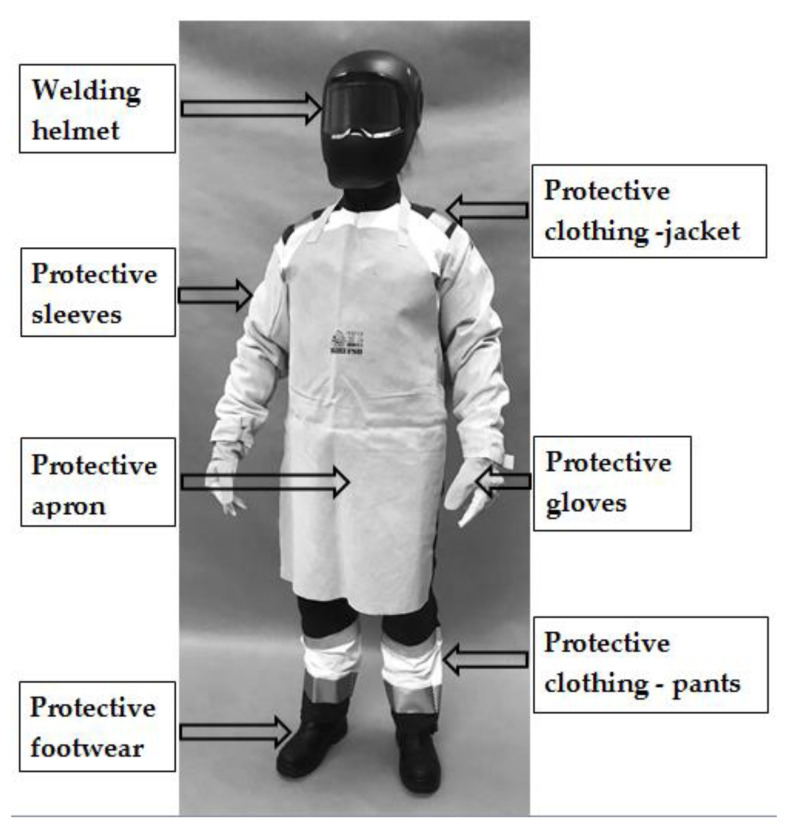
View of the subject wearing the welding PPE kit used in the study.

A complete welder’s outfit was selected for the present study on the methodology of determining dimensional allowances for PPE kits. The kit consisted of a ¾ length jacket, bib-and-brace overalls, safety footwear, protective gloves, welding helmet, as well as leather sleeves and apron providing additional protection against welding hazards. The constituent elements of the welding PPE kit used in the study are characterized in Table 1, while a photograph of a subject wearing the PPE kit is presented in Figure 3.

Anning was performed using a manual Artec 3D Eva scanner (Artec Group, Luxembourg, Luxembourg) with 0.1 mm accuracy and 0.2 mm resolution [43]. Ten replicated body scans of 1 volunteer young 30-year-old male by 4 operators (5th percentile due to 171 cm height) were performed in the study. The obtained 3D images were processed with MeshLab [44] and analyzed with CloudCompare software [45].

**Figure 4:**
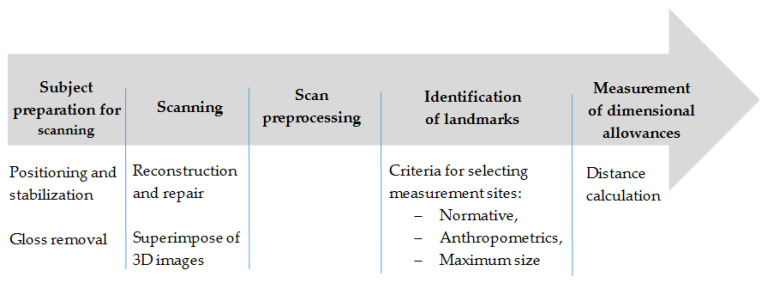
Flowchart of procedure for determining dimensional allowances for PPE using CloudCompare software.

The standard procedure for determining dimensional allowances consisted of five basic steps. The first step involved subject preparation (the subject needs to be positioned in the same, repeatable posture and the PPE surface must be matted). After scanning the subject (step two), the obtained image was preprocessed (scan repair and superposition of the images of subjects wearing underwear and PPE). Subsequently, measurement landmarks were identified (step four), according to the adopted criteria: normative, anthropometric, and, most importantly, the maximum silhouette size (step five). A flowchart of the general procedure for determining dimensional allowances is given in Figure 4. 

The detailed procedure of determining dimensional allowances for subjects wearing a complete welding PPE kit using geometric solids was implemented in CloudCompare software and consisted of the following steps:

- Subject preparation for scanning: model repair and cleaning–artifact removal (Edit/Segment); model positioning with respect to the software coordinate system (Edit/(Translate/Rotate)); generating a point cloud after processing (Navigation bar/Sample points on a mesh);
- Preliminary sizing to identify landmarks for collecting information about maximum dimensions (Tools/Segmentation/Cross-section);
- Generating a geometric solid–cylinder/cuboid (File/Primitive Factory);
- Superimposing the solid on 3D models of subjects wearing underwear and PPE (Edit/Translate/Rotate);
- A comparison of the dimensions of the solids and calculating differences in size between subjects wearing underwear and PPE, known as dimensional allowances.

**Figure 5:**
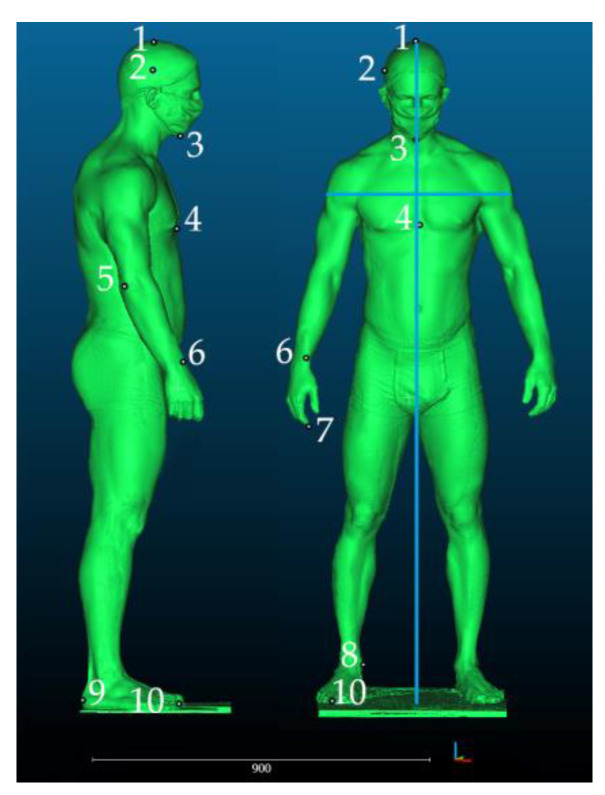
Anthropometric landmarks used for determining the maximum dimensions of the human body and auxiliary lines (blue) marking the width and height of geometric solids. 1–Vertex. 2—Eurion. 3—Gnathion. 4—Xyphoidale. 5—Radiale. 6—Stylion. 7—Daktylion. 8—Sphyrion. 9—Pternion. 10—Akropodion.

After preprocessing (repair and artifact removal), scans were imported to CloudCompare software. The next step involved the selection of anthropometric landmarks–external points demarcating the maximum dimensions of the human silhouette. At the same time, the distances between the landmarks constituted the minimum dimensions of geometric solids in which the silhouette could be inscribed. Figure 5 shows the selected landmarks. 

Cylinder dimensions were determined on the basis of the following maximum dimensions: in the *X* axis–the maximum distance between the shoulders (half of that distance is the cylinder radius), and in the *Y* axis–the maximum height of the human body, which is the height of the solid. In the case of the cuboid, the human silhouette was modeled using segments in three axes (*x*, *y*, and *z*); the base of the cuboid was defined by segments *x* (depth) and *z* (width), and its height by segment *y*.

**Figure 6:**
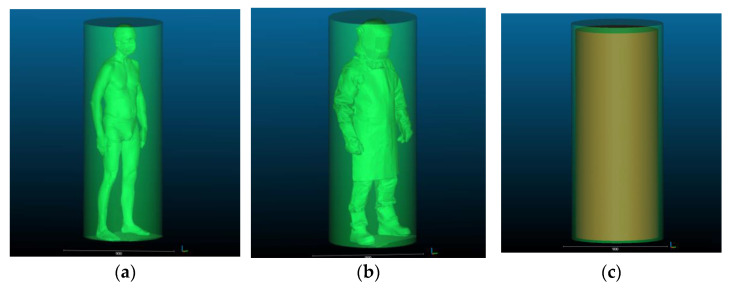
Human silhouette: (**a**) in underwear circumscribed by a cylinder; (**b**) in welding PPE–circumscribed by a cylinder; and (**c**) illustration of dimensional allowances for the welding PPE kit used in the study showing differences in the size of the human body in underwear and in PPE.

**Figure 7:**
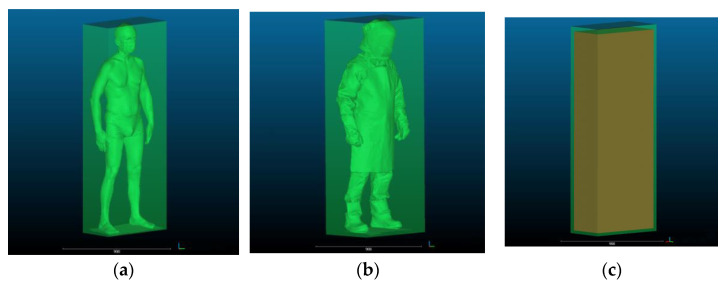
Human silhouette: (**a**) in underwear circumscribed by a cuboid; (**b**) in welding PPE–circumscribed by a cuboid; and (**c**) illustration of dimensional allowances for the welding PPE kit used in the study showing differences in the size of the human body in underwear and in PPE.

The human silhouette circumscribed by geometric solids corresponding to the actual shapes of confined and enclosed spaces found in work environments are given in Figure 6 and Figure 7. A view of dimensional allowances, i.e., size differences between solids circumscribing the silhouettes of the subject in underwear and in welding PPE is provided in Figure 6c and Figure 7c.

## 3. Results

The following dimensions were measured with the help of 3D scanning:

- the radius and height of the cylinder circumscribing the silhouette of the subject in underwear (*r*_u_, *h*_u_);
- the radius and height of the cylinder circumscribing the silhouette of the subject wearing PPE (*r*_PPE_, *h*_PPE_);
- the length of the edges of the cuboid circumscribing the silhouette of the subject in underwear (*x*_u_, *y*_u_*, z*_u_);
- the length of the edges of the cuboid circumscribing the silhouette of the subject wearing PPE (*x*_PPE_, *y*_PPE_*, z*_PPE_).

Measurements were conducted by four operators, each of whom performed 10 replicates. The obtained dimensions were used to calculate:

- volumes of the cylinders circumscribing the silhouette of the subject wearing underwear and PPE (*V*_C_u_*,V*_C_PPE_);
- volumes of the cuboids circumscribing the silhouette of the subject wearing underwear and PPE (*V*_Q_u_*,V*_Q_PPE_);
- base areas of the cylinders circumscribing the silhouette of the subject wearing underwear and PPE (*A*_C_u_*, A*_C_PPE_);
- base areas of the cuboids circumscribing the silhouette of the subject wearing underwear and PPE (*A*_Q_u_*, A*_Q_PPE_);
- differences and relative differences between all dimensions obtained for the silhouettes of the subject wearing underwear and PPE.

Four operators performed the measurements in 10 replicates each, and so means and standard deviations were calculated for each of the aforementioned values.

The numerical values given in Table 2 and Table 3 describe the geometric solids in which the silhouettes of individuals wearing underwear or a welding PPE kit can be inscribed. In turn, the absolute and relative differences in these values correspond to dimensional allowances for PPE. The actual values of dimensional allowances for PPE will be used for determining the minimum dimensions of confined workspaces, such as entry and exit openings, access openings, and manholes.

Table 4 and Table 5 contain what the authors consider the most important parameters describing cylinders and cuboids, i.e., base area and volume. The base area of the solids circumscribing the silhouette of the subject wearing PPE corresponds to the actual minimum area of access openings.

**Figure 8:**
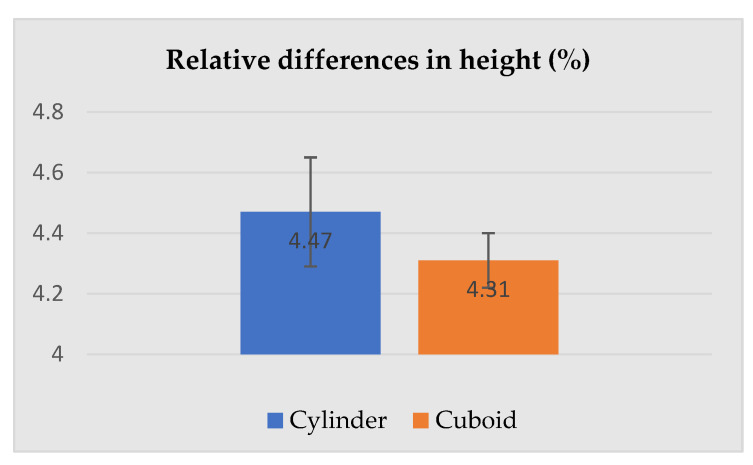
Relative differences in the height of the human silhouette circumscribed by a cylinder and cuboid attributable to PPE use.

**Figure 9:**
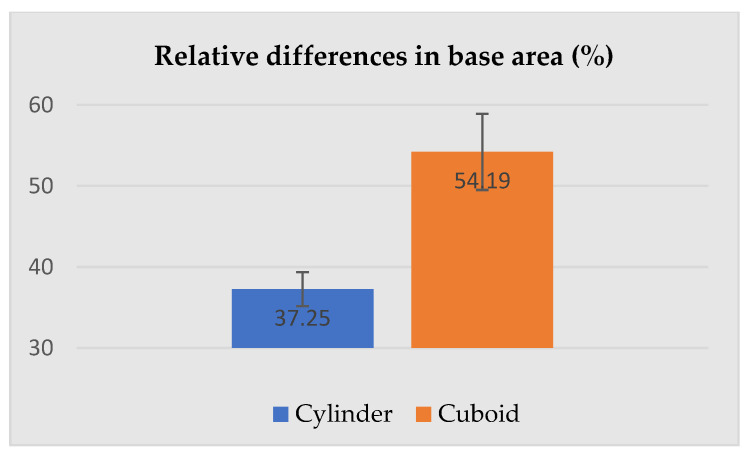
Relative differences in the base area of the human silhouette circumscribed by a cylinder and cuboid attributable to PPE use.

**Figure 10:**
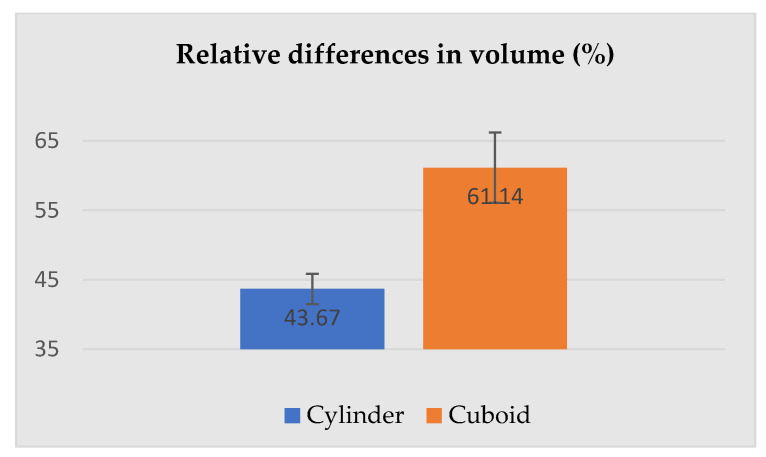
Relative differences in the volume of the human silhouette circumscribed by a cylinder and cuboid attributable to PPE use.

The dimensions presented above were used for the calculation of the relative differences (increase) between the selected dimensions of the geometric solids corresponding to the human subject wearing underwear and PPE. The most important parameters describing cylinders and cuboids, i.e., height, base area and volume are given in Figure 8, Figure 9 and Figure 10.

## 4. Discussion

The numerical values and differences given in Table 2, Table 3, Table 4 and Table 5 correspond to the dimensions of the scanned human silhouette and show relative changes in the height, base area, and volume of geometric solids circumscribing the subject in underwear and PPE. The numerical values given in Table 2 and Table 4 describe geometric solids in which the silhouettes of individuals wearing underwear or a welding PPE kit can be inscribed. In turn, absolute and relative differences in these values correspond to dimensional allowances for PPE. The actual values of dimensional allowances for PPE will be used for determining the minimum dimensions of confined workspaces, such as entry and exit openings, access openings, and manholes.

Table 3 and Table 5 contain the most important parameters describing cylinders and cuboids, i.e., base area and volume. The base area of the solids circumscribing the silhouette of a subject wearing PPE corresponds to the actual minimum area of access openings.

Figure 8, Figure 9 and Figure 10 present relative differences in the height, base area, and volume of the silhouettes circumscribed by cylinders and cuboids. Figure 8 shows that regardless of the geometric solid used to circumscribe the human silhouette (cylinder or cuboid), relative differences in the height of the solids constructed for the subject in underwear and PPE were similar, at 4.47 ± 0.18% for the cylinder and 4.31 ± 0.09% for the cuboid – the height was a common constant. In the case of the base area (see Figure 9), the relative change between the subject in underwear and PPE was much higher for a cuboid (54.19 ± 4.70%) than a cylinder (37.25 ± 2.10%).

The relative differences in height and the base area between the subjects in underwear and PPE were similar (higher for the cuboid), also the relative difference in volume (see Figure 10) 0 was significantly greater for the silhouette described by the cuboid (61.14 ± 5.04%) compared to the same silhouette described by the cylinder (43.67 ± 2.17%).

In the case of the human silhouette circumscribed by a cylinder, the use of PPE led to the following changes:

- an increase in cylinder radius (*r*) by 0.0513 m, reflecting an increase in the maximum distance between the shoulders by 2*r* = 0.1026 m*,* is the width allowance for PPE;
- an increase in cylinder height (*h*) by 0.0802 m, which is the height allowance for protective footwear and head protection devices.

In the case of the human silhouette circumscribed by a cuboid, the use of PPE led to the following changes:

- an increase in cuboid height (*y*) by 0.774 m, which is the height allowance added to the height of persons wearing PPE;
- an increase in cuboid width (*z*) by 0.1046 m, which is the width allowance;
- an increase in cuboid depth (*x*) by 0.1042 m.

Based on the obtained results, it can be stated that there is a correlation between changes in the human body dimensions and the most important solids’ parameters.

The differences in the so-called dimensional allowances determined in accordance with the proposed methodology can be used as data for the design of work environments. The purpose of using geometric solids was to show the importance and advantage of 3D spatial analysis over 2D. It was particularly useful when determining the dimensional allowances for width and depth. When determining dimensional allowances for the maximum body dimensions, the occurrence of different types of human silhouettes can be ignored. Moreover, the application of geometric solids for the spatial modeling of the human body directly shows the increase in the space occupied by a human subject wearing PPE. In practice, the determination of the minimum space occupied by workers in PPE will have an immediate influence on the safety of movement in confined spaces, decreasing the risk of getting stuck.

## 5. Conclusions

The work describes a study of the dimensional allowances which are a consequence of wearing personal protective equipment. In this case the allowances are treated as a factor in improving the safety of human interactions with the work environment. A 3D scanning method was proposed for determining dimensional allowances, which were modeled using geometric solids, i.e., a cylinder and a cuboid. These solids were selected because they corresponded to the human silhouette and the shapes of confined and enclosed spaces found in work environments (spaces with circular or rectangular cross-sections).

The study showed that the most important parameters defining the geometric solids modeling the human silhouettes are height, base area, and volume. It is impossible to select a single parameter as all of them are applicable in different situations: for instance, height for designing passage openings, volume for designing work zones, and base area for designing manholes. Based on the aforementioned parameters, solid dimensions, base area, and volume, it can be shown that a cylinder more accurately describes the area occupied by the human subject, as it exhibits a better fit to the human body as compared to a cuboid. However, the space occupied by humans needs to be described by both geometric solids in question (a cuboid and a cylinder) due to the varied spatial infrastructure of confined work environments. The present investigation is only a case study of a person moving in a work environment with limited space in a standing position. In further research, other human positions while performing tasks in the work environment should be included in the analysis, e.g., sitting and squatting. Then the minimum space necessary to perform work activities in a safe way will need to be defined using other solids, such as a sphere.

The results of the present analysis should be taken into account by professionals designing both occupational and non-occupational infrastructure. The main idea of the study was to point out to PPE designers that, e.g., protrusions can increase the space needed for work and may create additional difficulties and create a hazardous situation. In addition, engineers designing workspaces should be aware of the real external dimensions of a person with and without PPE as adding the total dimensional allowances resulting from PPE will make the workspace safe and more comfortable.

In the future, the results will be implemented in a new anthropometric atlas, namely, the Atlas of Human Measures, which is currently being developed at the Central Institute for Labor Protection–National Research Institute.

## Figures and Tables

**Table 1:** Characteristics of the personal protective equipment used for tests.

PPE Element	PPE Characteristics
Protective clothing (jacket and overalls)	- heat and flame resistance, symbols: A1, A2, B1, C1, E2, F1, according to EN ISO 11612:2015 [30]; - antistatic properties, according to EN 1149-5:2018 [31]; - electric arc resistance, according to EN 61482-2:2020 [32]; - small splashes of molten metal resistance and flame spread resistance, symbols: A1+A2, class 2, according to EN 11611:2015 [33]; - increased visibility (reflective tape, background material), class 1, according to EN ISO 20471:2013/A1:2016 [34];
Welding helmet with automatic welding filter	- eye and face protection against mechanical factors, symbol BT, according to EN 166:2001 [35]; - electrical insulation, according to EN 175:1997 - fire resistance, according to EN 175:1997 [36]; - heat transfer resistance, according to EN 175:1997 [36]; - fully adjustable head harness - four-step shade adjustment (8–12) - UV/IR protection, according to EN 379:2003+A1:2009 [37]; - response time: 0.1 ms (at 20 °C), according to EN 379:2003+A1:2009 [37].
Protective gloves	- mechanical resistance, symbols: 2121X, according to EN 388:2016+A1:2018 [38]; - heat protection, symbols: 411 × 4X, according to EN 407:2004 [39]; - dexterity: type B, according to EN 12477:2001 [40].
Safety footwear S3 class	- resistance to fire and molten metals, symbol WG according to EN ISO 20349-2:2017 [41]; - resistance to contact heat, symbol HRO, according to EN ISO 20345:2011 [42]; - anti-electrostatic properties: symbol A, according to EN ISO 20345:2011 [43]; - resistance to oils, symbol FO, according to EN ISO 20345:2011 [43]; - slip resistance on steel and ceramic surfaces, symbol SRC, according to EN ISO 20345:2011 [42]; - mechanical resistance (to puncture from the ground, resistance to impact and compression of footwear tips, energy absorption in the heel area, symbols: P, E, according to EN ISO 20345:2011 [42];
Protective apron and sleeves	- mechanical resistance and thermal resistance (protection against small splashes of molten metal, sparks, short-term contact with a flame, and radiant heat), symbols: A1, class 2, according to EN 11611:2015 [33] and EN ISO 11612:2015 [30].

**Table 2:** Dimensions of the human silhouette circumscribed by a cylinder as well as absolute and relative differences between the subject in underwear and PPE.

	Subject in Underwear		Subject Wearing PPE	
	*r*_u_	*h*_u_	*r*_PPE_	*h*_PPE_
Cylinder radius and height (m)	0.2991 ± 0.0008	1.7142 ± 0.0048	0.3504 ± 0.0022	1.7944 ± 0.0044
	*r*_PPE_–*r*_u_		*h*_PPE_–*h*_u_	
Differences between the subject in underwear and PPE (m)	0.0513 ± 0.0026		0.0802 ± 0.0034	
	Δ*r*		Δ*h*	
Relative differences between the subject in underwear and PPE (%)	14.64 ± 0.66		4.47 ± 0.18	

Notes: *r*_u_—the radius of the cylinder circumscribing the silhouette of the subject in underwear; *h*_u_—the height of the cylinder circumscribing the silhouette of the subject in underwear; *r*_PPE_—the radius of the cylinder circumscribing the silhouette of the subject wearing PPE; *h*_PPE_—the radius and height of the cylinder circumscribing the silhouette of the subject wearing PPE; (*r*_PPE_–*r*_u_)—differences of radius between the subject in underwear and PPE; (*r*_PPE_–*r*_u_)—differences of heights between the subject in underwear and PPE; Δ*r*—relative differences between the radius *r*_u_ and *r*_PPE;_ Δ*h*—relative differences between the heights *h*_u_ and *h*_PPE_.

**Table 3:** Dimensions of the human silhouette circumscribed by a cuboid as well as absolute and relative differences between the subject in underwear and PPE.

	Subject in Underwear			Subject Wearing PPE		
	*x*_u_	*y*_u_	*z*_u_	*x*_PPE_	*y*_PPE_	*z*_PPE_
Length of cuboid edges (m)	0.3588 ± 0.0099	1.7163 ± 0.0012	0.5977 ± 0.0013	0.4707 ± 0.0280	1.7936 ± 0.0017	0.7019 ± 0.0030
	*x*_PPE_–*x*_u_		*y*_PPE_–*y*_u_		*z*_PPE_–*z*_u_	
Differences between the subject in underwear and PPE (m)	0.1125 ± 0.0184		0.0774 ± 0.0016		0.1042 ± 0.0025	
	Δ*x*		Δ*y*		Δ*z*	
Relative differences between the subject in underwear and PPE (%)	23.78 ± 2.58		4.31 ± 0.09		14.85 ± 0.31	

Notes: *x*_u_—the length of the depth of the cuboid circumscribing the silhouette of the subject in underwear; *y*_u_—the length of the height of the cuboid circumscribing the silhouette of the subject in underwear; *z*_u_—the width of the width of the cuboid circumscribing the silhouette of the subject in underwear; *x*_PPE_—the length of the depth of the cuboid circumscribing the silhouette of the subject in PPE; *y*_PPE_—the length of the height of the cuboid circumscribing the silhouette of the subject in PPE; *z*_PPE_—the width of the width of the cuboid circumscribing the silhouette of the subject in PPE; (*x*_PPE_–*x*_u_)—differences of lengths between the subject in underwear and PPE; (*y*_PPE_–*y*_u_)—differences of heights between the subject in underwear and PPE; (*z*_PPE_–*z*_u_)—differences of depth between the subject in underwear and PPE Δ*x*—relative differences between lengths *x*_u_ and *x*_PPE_; Δ*y*—relative differences between heights *h*_u_ and *h*_PPE;_ Δ*z*—relative differences between widths *z*_u_ and *z*_PPE_.

**Table 4:** Base areas and volumes of the human silhouette circumscribed by a cylinder as well as absolute and relative differences between the subject in underwear and PPE.

	Subject in Underwear		Subject Wearing PPE	
	*A*_C_u_	*V*_C_u_	*A*_C_PPE_	*V*_C_PPE_
Cylinder base area (m^2^) and volume (m^3^)	0.2809 ± 0.016	0.4814 ± 0.036	0.3855 ± 0.044	0.6917 ± 0.070
	*A*_C_PPE_–*A*_C_u_		*V*_C_PPE_–*V*_C_u_	
Differences in cylinder base area (m^2^) and volume (m^3^) between subject in underwear and PPE	0.1046 ± 0.055		0.2102 ± 0.093	
	Δ*A*w		Δ*V*w	
Relative differences in cylinder base area and volume between subject in underwear and PPE (%)	37.25 ± 2.10		43.67 ± 2.17	

Notes: *A*_C_u_—base areas of the cylinders circumscribing the silhouette of the subject wearing underwear; *V*_C_u_—volumes of the cylinder circumscribing the silhouette of the subject wearing underwear; *A*_C_PPE_—base areas of the cylinders circumscribing the silhouette of the subject wearing PPE; *V*_C_PPE_—volumes of the cuboids circumscribing the silhouette of the subject wearing PPE; (*A*_C_PPE_–*A*_C_u_)—differences in base area between subject in underwear and PPE; (*V*_C_PPE_–*V*_C_u_)—differences in volume between subject in underwear and PPE; Δ*A*w—relative differences between *A*_C_u_ and *A*_C_PPE;_ Δ*V*w—relative differences between *V*_C_u_ and *V*_C_PPE_.

**Table 5:** Base areas and volumes of the human silhouette circumscribed by a cuboid as well as absolute and relative differences between the subject in underwear and PPE.

	Subject in Underwear		Subject Wearing PPE	
	*A*_Q_u_	*V*_Q_u_	*A*_Q_PPE_	*V*_Q_PPE_
Cuboid base area (m^2^) and volume (m^3^)	0.2141 ± 0.0057	0.3675 ± 0.0098	0.3303 ± 0.0184	0.5925 ± 0.0335
	*A*_Q_PPE_–*A*_Q_u_		*V*_Q_PPE_–*V*_Q_u_	
Absolute differences in cuboid base area (m^2^) and volume (m^3^) between subject in underwear and PPE	0.1162 ± 0.0129		0.2250 ± 0.0240	
	Δ*A*_Q_		Δ*V*_Q_	
Relative differences in cuboid base area and volume between subject in underwear and PPE (%)	54.19 ± 4.70		61.14 ± 5.04	

Notes: *A*_C_u_—base areas of the cuboid circumscribing the silhouette of the subject wearing underwear; *V*_C_u_—volumes of the cuboids circumscribing the silhouette of the subject wearing underwear; *A*_C_PPE_—base areas of the cuboid circumscribing the silhouette of the subject wearing PPE; *V*_C_PPE_—volumes of the cuboids circumscribing the silhouette of the subject wearing PPE; (*A*_C_PPE_–*A*_C_u_)—differences in base area between subject in underwear and PPE; (*V*_C_PPE_—*V*_C_u_)–differences in volume between subject in underwear and PPE; Δ*A*w—relative differences between *A*_C_u_ and *A*_C_PPE;_ Δ*V*w–relative differences between *V*_C_u_ and *V*_C_PPE_.

## Data Availability

The data presented in this study are available on request from the corresponding author.
